# Clinical and genetic features in autosomal recessive bestrophinopathy in Chinese cohort

**DOI:** 10.1186/s12886-024-03574-8

**Published:** 2024-07-24

**Authors:** Dongsheng Zhao, Victoria Y. Gu, Yafu Wang, Jie Peng, Jiao Lyu, Ping Fei, Yu Xu, Xiang Zhang, Peiquan Zhao

**Affiliations:** 1 Department of Ophthalmology, Xin Hua Hospital, Shanghai Jiao Tong University School of Medicine, Shanghai, China; 2 Johns Hopkins Bloomberg School of Public Health, Baltimore, MD USA

**Keywords:** Autosomal recessive bestrophinopathy, Anterior segment findings, Ultra-widefield scanning laser ophthalmoscopy, Laser peripheral iridotomy, *BEST1* mutation

## Abstract

**Purpose:**

To provide a genotype and phenotype characterization of the *BEST1* mutation in Chinese patients with autosomal recessive bestrophinopathy (ARB) through multimodal imaging and next-generation sequencing (NGS).

**Methods:**

Seventeen patients from 17 unrelated families of Chinese origin with ARB were included in a retrospective cohort study. Phenotypic characteristics, including anterior segment features, were assessed by multimodal imaging. Multigene panel testing, involving 586 ophthalmic disease-associated genes, and Sanger sequencing were performed to identify disease-causing variants.

**Results:**

Among 17 ARB patients, the mean follow-up was 15.65 months and average onset age was 30.53 years (range: 9–68). Best corrected visual acuity ranged from light perception to 0.8. EOG recordings showed a typically decreased Arden ratio in 12 patients, and a normal or slightly decreased Arden ratio in two patients. Anterior features included shallow anterior chambers (16/17), ciliary pronation (16/17), iris bombe (13/17), iridoschisis (2/17), iris plateau (1/17), narrow angles (16/17) and reduced axial lengths (16/17). Sixteen patients had multiple bilateral small, round, yellow vitelliform deposits distributed throughout the posterior pole, surrounding the optic disc. Initial diagnoses included angle-closure glaucoma (four patients), Best disease (three patients), and central serous chorioretinopathy secondary to choroidal neovascularization (CNV) (one patient), with the remainder diagnosed with ARB. Fourteen patients underwent preventive laser peripheral iridotomy, four of whom also received combined trabeculectomy and iridotomy in both eyes for uncontrolled intraocular pressure. One patient received intravitreal conbercept for CNV. Overall, 15 distinct disease-causing variants of *BEST1* were identified, with 14 (82.35%) patients having missense mutations. Common mutations included p. Arg255-256 and p. Ala195Val (both 23.68%), with the most frequent sites in exons 7 and 5.

**Conclusions:**

This study provides a comprehensive characterization of anterior segment and genetic features in ARB, with a wide array of morphological abnormalities. Findings are relevant for refining clinical practices and genetic counseling and advancing pathogenesis research.

## Introduction

Autosomal recessive bestrophinopathy (ARB, OMIM 611,809) was first described in a case report by Schatz et al. in 2006 [1]. It is an inherited retinal dystrophy (IRD) associated with biallelic variants in the bestrophin-1 gene (*BEST1*, OMIM *607,854). In 2008, Burgess et al. reported that ARB was both genotypically and phenotypically distinct from Best vitelliform macular dystrophy (BVMD, OMIM 153,700) [2]. However, ARB presents with a wide spectrum of fundus abnormalities [3] that notably overlap with many other IRDs, including BVMD, adult-onset vitelliform macular degeneration (OMIM 608,161), and autosomal dominant vitreoretinochoroidopathy (OMIM 193,220). This ambiguity presents a significant challenge for clinicians in determining the correct diagnosis and prognosis for ARB patients, which is crucial for early intervention or prevention. ARB is itself characterized by an extensive spectrum of fundus malformations, including multiple small, round, yellow lesions caused by vitelliform material deposits throughout the posterior pole, corresponding to multiple hyper-autofluorescence on fundus autofluorescence (FAF) [4–7]. However, the features of the anterior segment in ARB patients have been scarcely reported in the literature.

Since the first report of ARB, variable phenotypes have been observed and reported on, including diverse ages at onset, phenotypic expressions, and rates of disease progression. These variations occur not only between unrelated patients with the same mutation but also among individuals within the same family [8–14]. However, due to its rarity, a definitive panel of ARB’s clinical features, as well as knowledge of its progression and prognoses, remains underdeveloped. In this study, 19 patients from 17 unrelated families with ARB who were diagnosed by next-generation sequencing (NGS) underwent detailed comprehensive ophthalmic examinations. The purpose of this study was to report on ARB’s clinical and genotypic traits, as well as its progression and prognosis in the Chinese population.

## Methods

### Subjects and ethics statement

The research involving human subjects was approved by the Institutional Review Board of Xin Hua Hospital, affiliated to Shanghai Jiao Tong University School of Medicine (XHEC-D-2023-154). All routines adhered to the tenets of the Declaration of Helsinki. Written informed consent was obtained from all participants or their guardians for participation in the study and the potentially identifiable data presented in the tables.

### Clinical assessment

All participants diagnosed with ARB and their asymptomatic relatives were recruited from December 2016 to October 2020 in the outpatient department of the Xinhua Hospital of Jiaotong University, Department of Ophthalmology. Comprehensive clinical examinations were performed on all 19 patients from 17 non-consanguineous families, and included best-corrected visual acuity (BCVA), intraocular pressure (IOP, Goldmann tonometry), slit-lamp biomicroscopic ophthalmoscopy, dilated fundoscopy, ultra-widefield scanning laser ophthalmoscopy (Optos 200Tx; Optos, Dunfermline, UK), ultrasound biomicroscopy (UBM, MD-300 L; MEDA Co., Ltd., Tianjin, China), low-coherence interferometry (LenStar 900; Haag-Streit, Koeniz, Switzerland), swept-domain optical coherence tomography (SD-OCT; Heidelberg Engineering, Heidelberg, Germany), OCT angiography (OCTA, Optovue RTVue XR 100 Avanti; Fremont, CA, USA), electrooculography (EOG), and fluorescein angiography (FFA, Topcon TRC 501X; Topcon, Tokyo, Japan). Available family members were evaluated by BCVA, IOP, slit-lamp biomicroscopic ophthalmoscopy exam, dilated fundoscopy, fundus photography, SD-OCT, and EOG. Five patients had a history of laser peripheral iridotomy (LPI), trabeculectomy, and iridotomy.

### Genetic analyses

Peripheral blood samples were collected from all patients and available family members, and DNA was extracted from whole blood using the FlexiGene DNA Kit (Qiagen, Venlo, the Netherlands) according to the manufacturer’s protocol. The process was designed to include exon sequences from 586 genes, extending the exon area to 30 bp upstream and downstream, and reducing the impact of genomic reorganization. All genes were identified using OMIM, RetNet, and published literature associated with common inherited eye diseases (mRNA reference sequence: NM_001139443.1|NM_004183.3).

The sequence reads were aligned to the reference human genome (UCSC hg 38) using Burrows-Wheeler Aligner version 0.7.10 (BWA-MEM)15. The obtained sequence data were analyzed as described elsewhere15-18. Previously reported variants were identified using HGMD (professional version 2018.4), ClinVar (https://www.ncbi.nlm.nih.gov/clinvar/), and locus-specific databases. For variants that passed the initial filtration, Sanger sequencing was used to verify them within other family members.

## Results

### Clinical evaluation

A total of 17 patients from 17 unrelated families of Chinese origin with ARB, along with their available family members (*n* = 36), were recruited. The clinical details of these probands are summarized in Table 1. The mean follow-up time was 15.65 months, and the mean age at onset was 30.53 years (range, 9–68 years). Patients’ BCVA ranged from light perception to 0.8. EOG recordings showed a typically decreased Arden ratio in 12 of the affected individuals and a normal or slightly decreased Arden ratio in two patients (B II-1; K II-1); the other three patients did not undergo EOG. The alterations of the anterior and posterior segments of the eyes at the first visit are shown in Tables 2 and 3, respectively. Five patients were initially diagnosed with angle-closure glaucoma (ACG), three with BVMD, one with central serous chorioretinopathy secondary to choroidal neovascularization (CNV), and the others with ARB.

**Table 1:** Specific clinical materials of these probands with autosomal recessive bestrophinopathy (ARB)

Family	Patients	Status	Age/Sex	BCVA		IOP	(mmHg)	EOG	Initial	Therapy	Follow-up
			years	OD	OS	OD	OS		diagnosis		(months)
A	(II:1)	Proband	15/F	20/50	20/32	20	27.3	1.1/1.1	Best	LPI(OU)	38
B	(II:1)	Proband	11/M	20/25	20/500	23.3	23.5	1.4/1.2	Best	Conbercept	24
C	(II:1)	Proband	27/F	20/50	20/100	23.7	21.2	1.1/0.97	ARB	LPI(OU)	20
D	(II:1)	Proband	9/F	20/160	20/32	21	20.9	1.2/1.2	ARB	LPI(OD)	23
E	(II:1)	Proband	11/M	20/50	20/40	24.1	25.6	1.2/1.1	Best	LPI(OU)+Conbercept	26
F	(II:1)	Proband	68/F	20/40	20/63	16.9	17.4	1.2/1.0	ARB	-	36
G	(II:1)	Proband	58/F	20/50	20/32	22.3	24.6	1.1/1.2	ARB	LPI(OU)	8
H	(II:1)	Proband	13/M	20/32	20/25	16.2	15.4	1.1/1.2	ARB	-	6
I	(II:1)	Proband	33/F	20/32	20/40	17	19	1.0/1.1	ARB	LPI(OU)	5
J	(II:1)	Proband	24/M	20/50	20/100	23	20	1.1/1.0	ARB	LPI(OU)	9
K	(II:1)	Proband	26/M	20/100	20/80	24.1	22.3	1.4/1.4	CSC + CNV	LPI(OU)+Conbercept	12
L	(II:1)	Proband	38/F	20/100	20/125	21	24	1.2/1.3	ARB	LPI(OU)	23
M	(II:1)	Proband	36/F	20/160	20/100	22	24	1.1/1.1	ARB	LPI(OU)	26
N	(II:1)	Proband	18/F	20/200	20/160	16	14	NA	glaucoma	LPI(OU)+Trab + Iri	2
O	(II:1)	Proband	56/F	20/125	HM/BE	12	9	NA	glaucoma	LPI(OU)+Trab + Iri	4
P	(II:1)	Proband	40/F	20/50	20/400	12	14	NA	glaucoma	LPI(OU)+Trab + Iri	3
Q	(II:1)	Proband	36/F	20/80	20/200	8	10	1.2/1.2	glaucoma	LPI(OU)+Trab + Iri	1

ARB: autosomal recessive bestrophinopathy; CSC: central serous chorioretinopathy; CNV: choroidal neovascularization; F:female; LPI: laser peripheral iridotomy; M:male; Trab: trabeculecyomy; Iri: iridotomy;

**Table 2:** The alteration of anterior segmant of eyes in 17 probands with ARB at first visit

Family	AL(mm)		CCT(µm)		AD (mm)		Ciliary	Iris	Narrow angle
	OD	OS	OD	OS	OD	OS			
A	21.27	21.25	566	565	1.7	1.74	pronation	bombe	Slit-like
B	23.34	23.51	546	533	3.09	3.03	-	Iridoschisis	-
C	21.27	21.25	546	533	1.96	1.87	pronation	bombe	Slit-like
D	21	20.92	543	531	2.24	2.07	pronation	bombe	Slit-like
E	20.91	21.1	525	528	2.92	2.95	pronation	bombe	Slit-like
F	21.33	21.07	518	509	2.02	1.99	pronation	bombe	Slit-like
G	21.67	21.76	508	507	1.79	2.18	pronation	bombe	Slit-like
H	21.29	21.23	537	546	2.17	2.09	pronation	bombe	Slit-like
I	21.04	21.13	520	527	3.75	2.13	pronation	bombe + plateau	Slit-like
J	21.56	21.65	499	502	1.89	2.07	pronation	bombe	Slit-like
K	20.73	21.26	527	518	2.11	2.13	pronation	bombe	Slit-like
L	20.17	19.79	496	506	2.16	1.88	pronation	bombe	Slit-like
M	20.76	20.59	491	481	2.02	2.04	pronation	bombe	Slit-like
N	21.38	21.75	589	621	1.32	2.14	-	Iridoschisis	Slit-like
O	21.42	21.32	551	542	1.94	1.95	thin	flat	Slit-like
P	21.05	20.83	531	523	2.62	1.35	pronation	flat	Slit-like
Q	21.38	21.29	531	525	1.92	2.01	pronation	bombe	Slit-like

AD: aqueous depth; AL: axial length; CCT: cornea thickness;

**Table 3:** The alteration of posterior segmant of eyes with ARB at first visit

Family	IRF		SRF		Retinoschisis		CNV		The integrity of ELM		The position of yellowish subretinal deposits
	OD	OS	OD	OS	OD	OS	OD	OS	OD	OS	OU
A	(+)	(+)	(+)	(+)	(-)	(-)	(-)	(-)	(+)	(+)	Throughout the posterior pole
B	(+)	(+)	(+)	(+)	(-)	(-)	(+)	(+)	(+)	(-)	In the macula
C	(+)	(+)	(+)	(+)	(+)	(+)	(-)	(-)	(+)	(+)	Throughout the posterior pole
D	(+)	(+)	(+)	(+)	(+)	(+)	(+)	(+)	(-)	(-)	Throughout the posterior pole
E	(+)	(+)	(+)	(+)	(+)	(+)	(+)	(+)	(-)	(-)	Throughout the posterior pole
F	(+)	(+)	(+)	(+)	(-)	(-)	(-)	(-)	(+)	(+)	Throughout the posterior pole
G	(+)	(+)	(+)	(+)	(-)	(-)	(-)	(-)	(+)	(+)	Throughout the posterior pole
H	(+)	(+)	(+)	(+)	(-)	(-)	(-)	(-)	(+)	(+)	Throughout the posterior pole
I	(+)	(+)	(+)	(+)	(-)	(-)	(-)	(-)	(+)	(+)	Throughout the posterior pole
J	(+)	(+)	(+)	(+)	(+)	(+)	(-)	(-)	(+)	(+)	Throughout the posterior pole
K	(+)	(+)	(+)	(+)	(+)	(+)	(+)	(-)	(+)	(+)	Throughout the posterior pole
L	(+)	(+)	(+)	(+)	(+)	(+)	(-)	(-)	(+)	(+)	Throughout the posterior pole
M	(+)	(+)	(+)	(+)	(-)	(-)	(-)	(-)	(+)	(+)	Throughout the posterior pole
N	(+)	(+)	(+)	(+)	(+)	(+)	(-)	(-)	(+)	(+)	Throughout the posterior pole
O	(+)	(+)	(+)	(+)	(+)	(+)	(-)	(-)	(+)	(+)	Throughout the posterior pole
P	(+)	(+)	(+)	(+)	(+)	(+)	(-)	(-)	(+)	(+)	Throughout the posterior pole
Q	(+)	(+)	(+)	(+)	(+)	(+)	(-)	(-)	(+)	(+)	Throughout the posterior pole

IRF: intraretinal fluid; SRF: subretinal fluid; CNV: choroidal neovascularization; ELM: external limiting membrane; OD: right eye; OS: left eye; OU: both eyes

**Fig. 1:**
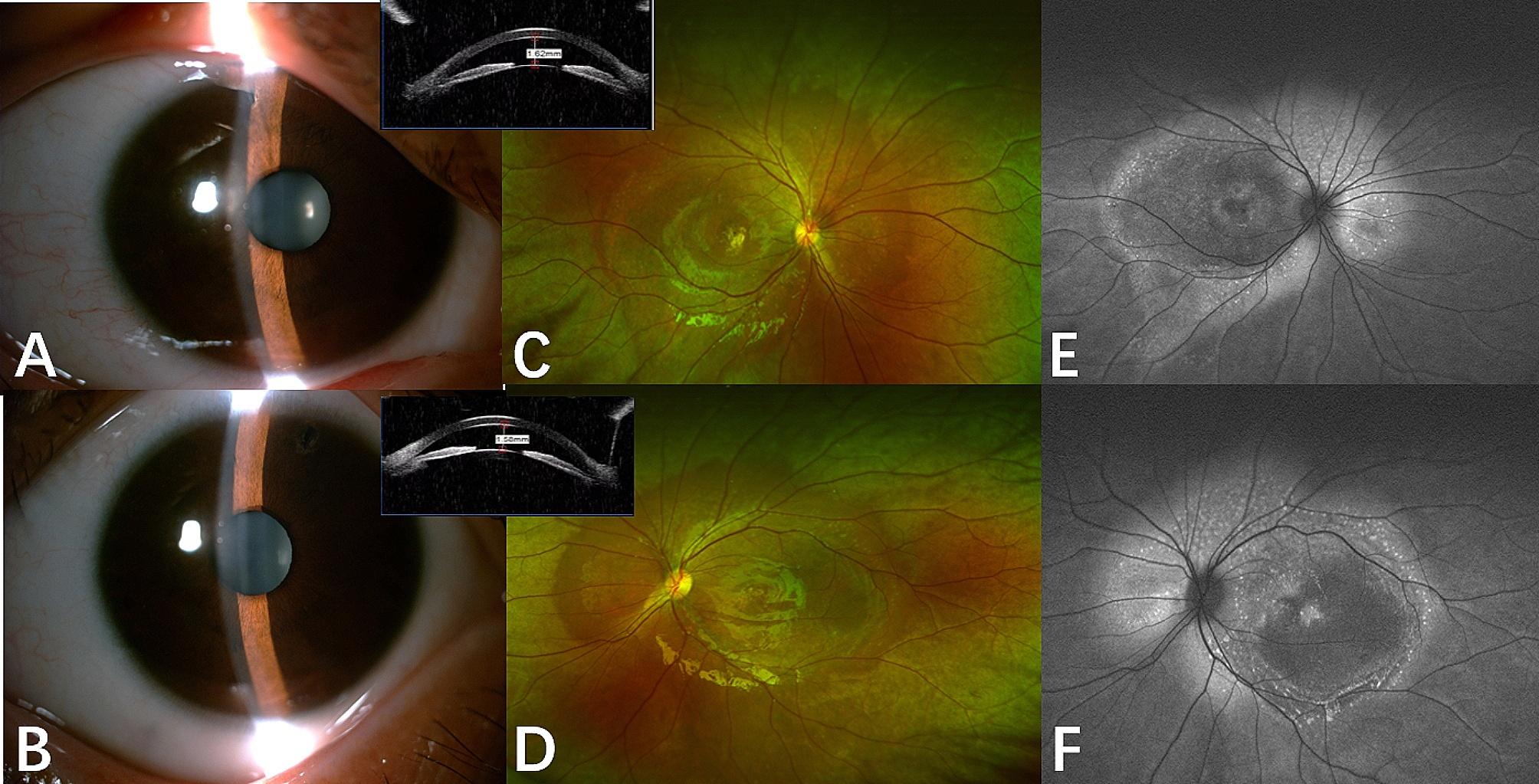
Multimodal imaging of the proband (II:1) from family B, harboring mutations p. Gln256Arg at baseline. Anterior features included shallow anterior chambers **(A, B)**. Fundus photographs show multiple small, round, yellow vitelliform deposits throughout the posterior pole surrounding the optic disk **(C and D)**, corresponding to central hyper-autofluorescence on fundus autofluorescence **(E and F)**

Anterior features included shallow anterior chambers (18/19),, ciliary pronation (18/19), iris bombe (14/19), iridoschisis (2/19), iris plateau (1/19), narrow angles (18/19) and reduced axial lengths (18/19) of ARB patients with multiple small, round, yellow vitelliform deposits throughout the posterior pole surrounding the optic disk in both eyes (18/19). Thirteen patients underwent preventive LPI, of whom five also had combined trabeculectomy and iridotomy in both eyes because of the uncontrolled IOP. One patient underwent intravitreal injection of conbercept (Chengdu Kanghong Biotechnologies Co. Ltd., ChengDu, China) because of central serous chorioretinopathy secondary to CNV. The anterior and fundus imaging were shown in Fig. 1.

### Genetic analyses

**Fig. 2:**
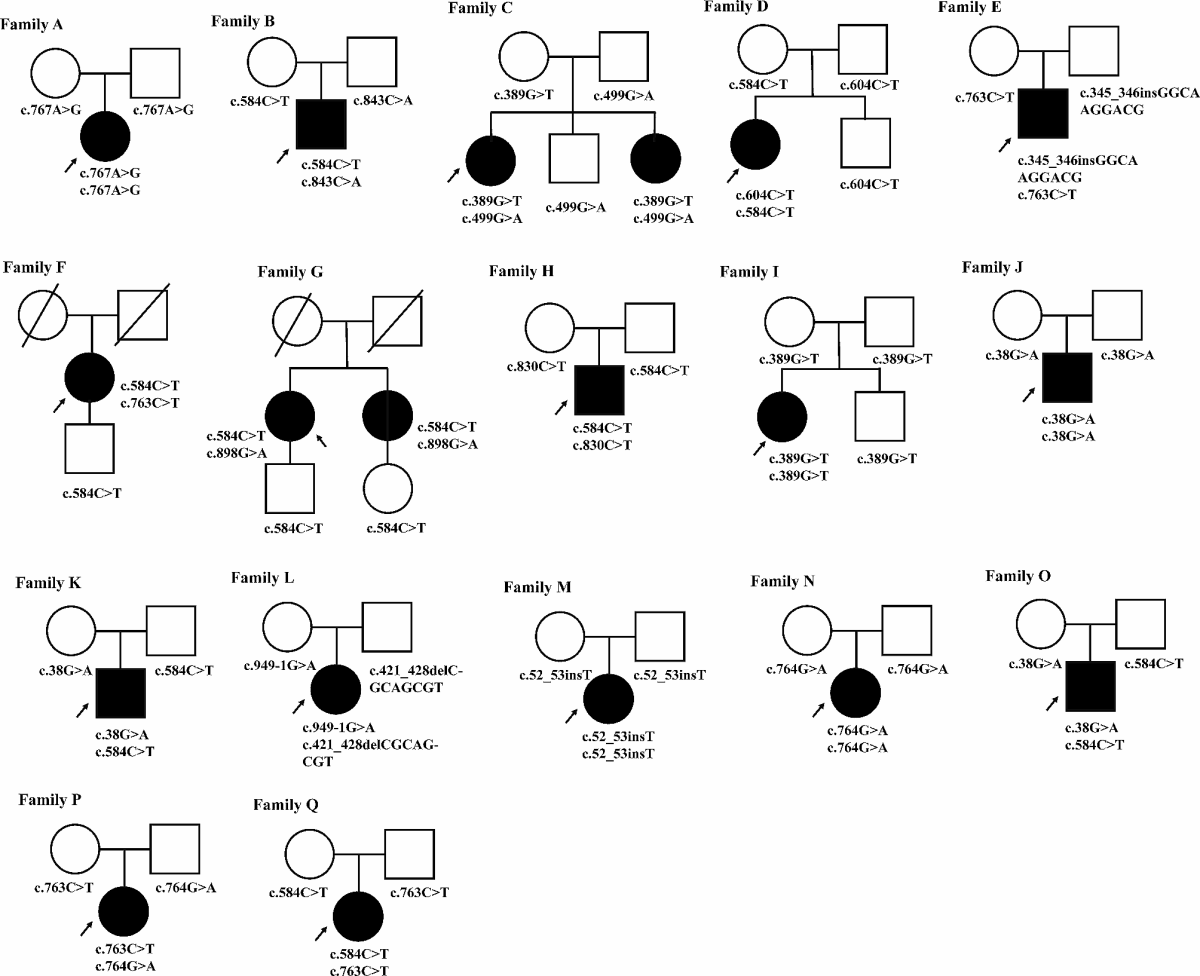
Pedigrees of the 17 families **(A–Q)** in this research with *BEST1* mutations. Filled symbols signify *ARB* patients. Unfilled symbols represent unaffected family members. Arrows: probands. Pluses: wild-type alleles; Square: male individuals; Circle: female individuals

Sixteen distinct *BEST1* variants were identified in this cohort (Fig. 2), including 12 missense (75%), one splice site (6.25%), and three frameshift (18.75%) variants (Table 4). Based on the 2015 American College of Medical Genetics’ (ACMG’s) standards and guidelines for the interpretation of sequence variants, 13 variants were determined to be pathogenic, while c.767 A > G and c.843 C > A were deemed likely pathogenic. Multiple orthologous sequence alignment revealed that subsequent codons 256 and 281 of *BEST1* were highly conserved among species, suggesting that any mutation at these codons would have a deleterious effect. Of the 17 patients in this study, 13 (76.47%) carried missense mutations. The most frequent was c.584 C > T (p. Ala195Val), found in 8 out of 34 alleles, with the most frequently mutated sites located in exons 5 (10/34 alleles) and 7 (12/34 alleles). All mutations identified in our cohort were clustered in the intracellular (67.65%, 23/34) and transmembrane regions (32.35%, 11/34). No electrophysiological or clinical abnormalities were identified in any of the heterozygotes.

**Table 4:** *BEST1* variants identified in this cohort of patients

Family Patient	Nucleotide Change	Amino Acid Change	Mutation Type	Exon/Intron	Localization	ACMG category
A-1	c.767A > G	p.Gln256Arg	Missense	EX7	transmembrane region	LP
	c.767A > G	p.Gln256Arg	Missense	EX7	transmembrane region	LP
B-1	c.584C > T	p.Ala195Val	Missense	EX5	intracelluar	P
	c.843C > A	p.Phe281Leu	Missense	EX7	transmembrane region	LP
C-1	c.389G > T	p.Arg130Leu	Missense	EX4	intracelluar	P
	c.499G > A	p.Glu167Lys	Missense	EX5	intracelluar	P
D-1	c.584C > T	p.Ala195Val	Missense	EX5	intracelluar	P
	c.604C > T	p.Arg202Trp	Missense	EX5	intracelluar	P
E-1	c.345_346insGGCAAGGACG	p.Glu115GlufsX120	frameshift	EX4	intracelluar	P
	c.763C > T	p.Arg255Trp	Missense	EX7	transmembrane region	P
F-1	c.584C > T	p.Ala195Val	Missense	EX5	intracelluar	P
	c.763C > T	p.Arg255Trp	Missense	EX7	transmembrane region	P
G-1	c.584C > T	p.Ala195Val	Missense	EX5	intracelluar	P
	c.898G > A	p.Glu300Lys	Missense	EX7	intracelluar	P
H-1	c.584C > T	p.Ala195Val	Missense	EX5	intracelluar	P
	c.830C > T	p.Thr277Met	Missense	EX7	transmembrane region	P
I-1	c.389G > T	p.Arg130Leu	Missense	EX4	intracelluar	P
	c.389G > T	p.Arg130Leu	Missense	EX4	intracelluar	P
J-1	c.38G > A	p.Arg13His	Missense	EX2	intracelluar	P
	c.38G > A	p.Arg13His	Missense	EX2	intracelluar	P
K-1	c.584C > T	p.Ala195Val	Missense	EX5	intracelluar	P
	c.38G > A	p.Arg13His	Missense	EX2	Intracellular (N-terminal)	P
L-1	c.421_428delCGCAGCGT	p.Arg141Glnfs88	frameshift	EX3	intracelluar	P
	c.949-1G > A		Splicing	EX8	intracelluar	P
M-1	c.52_53insT	p.Ser19Phefs153	frameshift	EX2	intracelluar	P
	c.52_53insT	p.Ser19Phefs153	frameshift	EX2	intracelluar	P
N-1	c.764G > A	p.Arg255Gln	Missense	EX7	transmembrane region	P
	c.764G > A	p.Arg255Gln	Missense	EX7	transmembrane region	P
O-1	c.584C > T	p.Ala195Val	Missense	EX5	intracelluar	P
	c.38G > A	p.Arg13His	Missense	EX2	intracelluar	P
P-1	c.763C > T	p.Arg255Trp	Missense	EX7	transmembrane region	P
	c.764G > A	p.Arg255Gln	Missense	EX7	transmembrane region	P
Q-1	c.584C > T	p.Ala195Val	Missense	EX5	intracelluar	P
	c.763C > T	p.Arg255Trp	Missense	EX7	transmembrane region	P

P: Pathogenic; LP: Likely pathogenic

## Discussion

The findings of this study provided several valuable insights into the clinical management and prognosis of ARB patients. Firstly, the prognosis of ARB patients varied significantly due to the differing timelines and approaches of therapeutic interventions. Remarkable angle alterations were observed in all patients except one. Ten patients underwent preventive LPI in the early stages due to abnormal ciliary bodies or irises and remained stable until the final follow-up. However, one patient (O II-1), who was initially diagnosed with ACG and underwent LPI, trabeculectomy, and iridotomy at other hospitals, experienced a worse prognosis in one eye. Conversely, three patients (N II-1, P II-1, and Q II-1) who were also initially diagnosed with ACG and who underwent LPI, trabeculectomy, and iridotomy at other hospitals remained stable until the final diagnosis. These varied prognoses underscore the importance of precise and early diagnosis and intervention for patients with ARB. Secondly, multi-modal imaging of the fundus revealed a diverse range of phenotypic presentations. In particular, CNV was detected in three patients by OCTA, while only one received an intravitreal injection of conbercept. Thirdly, the misdiagnosis rate of ARB was relatively high, likely attributable to the diversity of ocular phenotypes, ranging from isolated or widespread vitelliform retinal degeneration to an abnormal anterior chamber. Moreover, the EOG Arden ratio of ARB patients ranged from normal to markedly reduced.

### Phenotypic presentations

ARB exhibits a broad spectrum of clinical presentations, encompassing a range of unusual features and certain specific similarities in the anterior segments. These include hyperopia, shallow anterior chambers, ciliary pronation, iris bombe, iridoschisis, iris plateau, narrow anterior chamber angles, and reduced axial lengths (AL). The proportion of ARB patients developing angle-closure glaucoma (ACG) due to narrow anterior chamber angles has been reported to be up to 50%, potentially leading to further vision loss [2]. In the present study, 16 of 17 patients (94.11%) had narrow angles, and four patients (26.3%) had ACG with poor visual acuity. While the reasons behind these ocular abnormalities remain unknown, the transcription factors OTX2, microphthalmia-associated transcription factor (MITF), and cone-rod homeobox protein have been shown to modulate ocular developments via *BEST1* expression [15]. Notably, OTX2 and MITF have been reported to play a role in the differentiation of anterior segment formation, [16, 17] necessitating preventive LPI for ARB patients with a high risk of ACG.

Authors of this study hypothesized that preventive LPI was performed earlier in patients with ARB than in those with ACG. In this cohort, 14 patients received LPIs because of slightly elevated IOP and ocular abnormalities such as shallow anterior chambers, narrow anterior chamber angles, and reduced AL. However, the results of LPI for lowering IOP may be limited in some ARB patients during long-term follow up [18] due to dysgenesis of the anterior segment, including the dysplastic trabecular meshwork, a shallow anterior chamber, and narrow anterior chamber angles. Of note, Boon et al. suggested that patients with ARB may receive topical antiglaucomatous medication to reduce the IOP further [18], an approach that was adopted by clinicians for all patients in the present study. Four patients further underwent trabeculectomies and iridotomies to control IOP. The efficacy of early LPI in patients with ARB and the associated effects of various anti-glaucoma surgeries (e.g., trabeculectomy, iridotomy, and glaucoma valve implantation) should be further investigated in additional studies.

The first account of ARB by Burgess et al. in 2008 described the typical fundus appearance as yellowish with subretinal deposits scattered over the posterior pole, subretinal fluid accumulation beneath the macula, and cystoid macular edema [2]. These features differed from the extramacular and multi-vitelliform lesions seen in some cases of Best disease. To supplement this characterization, the present study reports retinoschisis in 10 probands, with patients from Family B presenting with vitelliform lesions and fibrotic CNV localized to the macula alone. This comprehensive evaluation of disease progression involving the monitoring of multiple lesions over the posterior pole was only made possible by advanced fundus imaging technology, specifically ultra-widefield scanning laser ophthalmoscopy. The numerous subretinal deposits localized to this area are currenly believed to reflect higher *BEST1* expression in the peripheral retinal pigment epithelium (RPE), compared to the macula [19]. Moreover, intraretinal edema is believed to be commonly observed in patients with ARB as *BEST1* mutations likely affect the differentiation of bipolar cells following *OTX* and *MITF* alterations in the *BEST1* promoter [15, 20, 21]. The fundus was also observed to undergo continuous changes despite the administration of oral medications and preventive LPI throughout the follow-up period [18].

A pronounced reduction or complete absence of the EOG light rise has been recognized as a defining characteristic of ARB [3]. In this study, 12 patients with a typical ARB-appearing fundus presented with a markedly decreased EOG Arden ratio. However, patient B II-1, who had lesions confined to the macula, showed only a slight reduction in the EOG Arden ratio. The Arden ratio measures the ratio of the light peak to the dark trough in an electro-oculogram (EOG) and serves as an indicator of retinal pigment epithelium function. The extent of the decreased EOG Arden ratio is believed to reflect the function of the Cl⁻ channel (BEST-1), with a greater number of vitelliform lesions scattered over the posterior pole indicating more severe impairment of BEST-1 function [3].

While indocyanine green angiography and FAF were once the gold standard for diagnosing CNV, [22] OCTA has revolutionized this procedure as a non-invasive, rapid technique for analyzing the morphology of the four layers [23] and visualizing CNV or confirming its presence together with hyporeflective vitelliform material [24]. OCTA has also previously been used to show vascular abnormalities that occurred in ARB and identified the vitelliform masking of CNV [25]. In the present study, three patients with CNV were followed up with for nearly 1 year with OCTA, which revealed distinct morphological changes at each visit.

### Genotypic analyses

ARB has wide phenotypic heterogeneity that can overlap with the clinical features of many other IRDs. Genotypic analysis has been instrumental in providing evidence to support clinical diagnoses and evolving disease risk for future patients. The present study identified 15 *BEST1* mutations, with a significant concentration (64.71%) in exons 5 and 7, suggesting these regions are critical hotspots for ARB mutations. Missense mutations (75%) were the most prevalent, which is consistent with previously reported findings [5, 12, 13]. Mutations c.763 C > T (p.Arg255Trp) and c.584 C > T (p.Ala195Val) are the most commonly reported among ARB patients of Chinese and Japanese origin; [5, 13] consistent with this, c.584 C > T was the most frequent mutation identified in this study. Furthermore, mutation c.763 C > T (p.Arg255Trp) was present in only 23.53% of the patient cohort. The analogous amino acid change c.764G > A (p.Arg255Gln) accounted for 11.76% of the mutations, while the adjacent amino acid mutation c.767 A > G (p.Gln256Arg) comprised 5.88%. Therefore, a total of 26.47% (9/34) of mutations occurred at the p.Arg255-256 site, which coincides with the location of c.584 C > T (p.Ala195Val). These results support the reported finding that the mutations p.Arg255-256 and p.Ala195Val are prevalent in ARB patients of Chinese origin.

Of these mutations, c.584 C > T, c.38G > A, c.763 C > T, and c.830 C > T have been previously identified not only in ARB but also in BVMD and/or Stargardt macular dystrophy, and retinitis pigmentosa [19, 26]. Conversely, c.898G > A has only been observed in cases of BVMD, and c.345_346insGGCAAGGACG has only been documented in cases of multifocal vitelliform dystrophy [27, 28]. No clinical or electrophysiological abnormalities were identified in any heterozygotes in the present study. These findings collectively illustrate the high genetic heterogeneity of *BEST1*.

The study has a number of limitations, including its comparatively small sample size. Additionally, experimental methods would aid in the confirmation of the pathogenic effects of the newly identified variants. Further studies are also needed to elucidate the differences between the molecular mechanisms of ARB and BVMD.

In conclusion, this study presents a broad range of ocular morphologic abnormalities identified in ARB cases using multimodal imaging, providing novel insights into the clinical and genotypic characteristics of ARB in patients of Chinese origin.

## Data Availability

The datasets used and/or analyzed during the current study are available from the author P.Z on reasonable request.

## Abbreviations

ACG: angle-closure glaucoma
ARB: autosomal recessive bestrophinopathy
BCVA: best-corrected visual acuity
BVMD: best vitelliform macular dystrophy
CNV: choroidal neovascularization
EOG: electrooculography
FFA: fluorescein angiography
IOP: intraocular pressure
LPI: laser peripheral iridotomy
MITF: microphthalmia-associated transcription factor
NGS: next-generation sequencing
OCTA: OCT angiography
SD-OCT: swept-domain optical coherence tomography
UBM: ultrasound biomicroscopy
